# The Nursing Practice Environment and Job Satisfaction, Intention to Leave, and Burnout Among Primary Healthcare Nurses: A Cross-Sectional Study

**DOI:** 10.3390/nursrep15070224

**Published:** 2025-06-21

**Authors:** Pedro Lucas, Élvio Jesus, Sofia Almeida, Patrícia Costa, Paulo Cruchinho, Gisela Teixeira, Beatriz Araújo

**Affiliations:** 1 Center for Interdisciplinary Research in Health (CIIS), Faculdade de Ciências da Saúde e Enfermagem, Universidade Católica Portuguesa, Rua de Diogo Botelho, 4169-005 Porto, Portugal; ejesus@ucp.pt (É.J.); ssalmeida@ucp.pt (S.A.); baraujo@ucp.pt (B.A.); 2 Nursing Research Innovation and Development Centre of Lisbon (CIDNUR), Nursing School of Lisbon, 1600-190 Lisbon, Portugal; patriciacosta@esel.pt (P.C.); pjcruchinho@esel.pt (P.C.); gteixeira@esel.pt (G.T.); 3 Nursing Administration Department, Nursing School of Lisbon, 1600-190 Lisbon, Portugal

**Keywords:** burnout, turnover, job satisfaction, work environment, primary healthcare, nursing

## Abstract

**Background:** The nursing practice environment significantly influences nurses’ job satisfaction, turnover, and burnout; therefore, it is essential to promote favorable environments to ensure the retention of qualified professionals. Improving the nursing practice environment is a low-cost organizational strategy associated with satisfaction, retaining professionals, and reducing burnout. The aim of this study was to assess the relationship between the nursing practice environment and job satisfaction, turnover, and burnout among primary healthcare nurses in Portugal. **Methods:** A descriptive, cross-sectional, and correlational study was carried out based on data from the RN4CAST Portugal Project. The Nurse Survey Instrument (Core Nurse Survey) of the RN4Cast Project (2018) was used for data collection. The sample consisted of 1059 nurses from fifty-five health center groups in mainland Portugal, fifteen health centers in the Autonomous Region of Madeira, and six health centers in the Autonomous Region of the Azores. **Results:** Primary healthcare nurses in Portugal rated the nursing practice environment as unfavorable or mixed, with an average ($\bar{x}$) of 2.5 (standard deviation (SD) = 0.4), which is associated with lower job satisfaction, with an average of 2.0 (SD = 0.4), moderate intention to leave, with 40.3%, and low levels of burnout, with an average of 1.6 (SD = 0.8). There was also a negative correlation between the nursing practice environment and burnout (r = −0.28) and its dimensions. Emotional exhaustion (r = −0.35) represents the individual dimension of stress and physical exhaustion, corresponding to feelings regarding the depletion of emotional and physical resources, depersonalization (r = −0.18) represents the interpersonal context dimension of burnout, and a lack of personal accomplishment (r = −0.15) represents the self-assessment dimension of burnout and refers to feelings of incompetence and a lack of confidence and self-efficacy at work. **Conclusions:** The quality of the work environment is associated with greater job satisfaction and a reduction in burnout. For this reason, improving the work environment has therefore been associated with increased job satisfaction and reduced burnout among primary healthcare nurses, promoting nurse retention and the well-being of healthcare teams.

## 1. Introduction

In Portugal, studies evaluating the nursing practice environment and its implications are scarce [1]. Nursing in Portugal is associated with high levels of stress due to insufficient staffing in the workplace, which leads to constant overload and pressure on professionals [2]. The poor recognition of nursing professionals’ skills and experience due to the lack of improvements in salaries and the increase in competition also contributes to professional dissatisfaction.

The nursing practice environment should be one of the main concerns of healthcare systems, as it is essential for preventing nursing shortages and guaranteeing the safety of the care provided [2,3]. Creating favorable nursing practice environments improves nurse retention and facilitates the quality of patient care [4]. The study by Twigg and McCullough [4] identifies strategies such as enabling work environments, shared governance structures, nurse autonomy, professional development, leadership support, adequate staffing, and collegial relationships within the healthcare team as being effective in creating such environments. Improving the working environment is therefore a fundamental goal to guarantee the effectiveness of the care provided and promote motivation in nurses [5].

Positive and healthy work environments are defined by the International Council of Nurses as contexts that favour excellence and the adequate performance of professionals [6]. They ensure the health, safety, and well-being of professionals, promote and encourage the provision of quality care, and improve the motivation, productivity, and performance of both employees and organizations [6].

Positive and healthy working environments influence and affect nurses and are therefore a dimension of the utmost importance for healthcare organizations [7].

The complex social and professional contexts in which nurses practice, and where the need for professionals is continuous, are called professional practice environments.

The nursing practice environment is made up of a set of characteristics, both concrete and abstract, of an organization that are interrelated with its processes and structure and are perceived by nurses as factors that can facilitate or hinder their professional practice [5,8,9].

A favorable practice environment is associated with better nursing outcomes [10], including higher job satisfaction and retention rates [11] and greater adherence to evidence-based practices [12].

The nursing practice environment is the factor that has the greatest impact and influence on nursing results and perceptions of the quality of care provided [9]. In the current context, due to the global crisis that has affected the world in recent years, these issues have gained even greater relevance [5]. According to different authors, the nursing practice environment is associated with multiple variables, which can be categorized as external or internal to the nurse [13,14]. It is recognized that the nursing practice environment can have an impact on nurses’ job satisfaction and intentions to leave [9].

Job satisfaction is a global concern. It is important to improve the quality of care provided and promote an appropriate working environment in healthcare organizations, and the absence of job satisfaction among nurses can compromise these issues, which in turn can affect patient satisfaction [15].

In addition, high rates of burnout pose a challenge not only to nurse retention but also risks to client safety and the quality of care provided, consequently resulting in decreased client satisfaction [7].

Burnout can use up nurses’ internal resources to cope with the demands of the job and the complexity of the care they must provide [16]. Burnout is defined as a psychological syndrome involving emotional exhaustion, depersonalization, and a diminished sense of professional accomplishment that arises in response to prolonged exposure to interpersonal stressors in the workplace [17]. This syndrome has an impact on nurses’ mental health and well-being and can jeopardize productivity, performance, and the quality of care provided [18]. Nantsupawat et al. [19] state that environmental factors are the strongest predictors of burnout. Employees’ perceptions of the work environment can manifest themselves through concern about working conditions, and burnout is seen as a reaction to that environment in terms of emotional exhaustion. In addition to environmental factors, the reason for choosing a profession was also identified as a personal factor that predicts burnout. As the reason for choosing a profession can contribute to attitudes in the workplace, this can be positively related to lower rates of burnout among professionals [20].

Stress factors resulting from inadequate staffing levels due to inadequate management are important factors in job satisfaction, with the likely consequence of burnout [21,22]. Poor human resource management decreases staff motivation, which leads to feelings of depersonalization, as well as job dissatisfaction. Improving the nursing practice environment to ensure that professionals feel safer and have adequate resources to carry out their duties is related to improved outcomes for clients [18,19].

There is little knowledge about the nursing practice environment in the context of primary healthcare [1,9,23,24], which has a negative impact on the expansion and valorization of nursing in this area [1,9,25].

Another issue affecting the nursing profession in Portugal was the non-recognition of clinical nursing specializations in nursing careers, which changed after data were collected for this study, which may be a conditioning factor for professionals’ motivation and intention to leave.

No study has been carried out in primary healthcare in Portugal on the work environment, job satisfaction, turnover, and burnout of nurses, which justifies the importance of this study.

The aim of this study was therefore to assess the relationship between the nursing practice environment and job satisfaction, turnover, and burnout among primary healthcare nurses in Portugal.

## 2. Materials and Methods

### 2.1. Study Design and Setting

This study, set in the context of primary healthcare, is part of the RN4Cast Project in Portugal (2018), which replicates the international RN4Cast study [26,27]. This is a descriptive, cross-sectional, and correlational study, whose aim was to assess the relationship between the nursing practice environment and the job satisfaction, turnover, and burnout of nurses in primary healthcare in Portugal. The STROBE guidelines [28] were applied.

### 2.2. Study Population and Eligibility Criteria

This study’s inclusion criteria were nurses with any type of labor contract who provide care in the primary healthcare services of the Portuguese National Health Service, on the mainland or in the autonomous regions of Madeira and the Azores.

A total of 1113 nurses answered the questionnaire. However, 54 responses were excluded for having incomplete or incorrect data, according to the criteria previously defined for inclusion. As inclusion criteria, nurses had to provide direct care to clients and have the professional category of nurse or nurse specialist, regardless of their employment relationship with the healthcare organization, during the data collection period. Thus, the final sample consisted of 1059 valid participants.

The sample consisted of 1059 nurses with different types of employment contracts in primary healthcare in the National Health Service. The nurses work in the 55 health center groups in mainland Portugal, 15 health centers in the Autonomous Region of Madeira, and 6 health centers in the Autonomous Region of the Azores. The sample size corresponds to what Marôco [29] advocates, consisting of between five and ten participants per item. Although no formal power analysis was carried out for this study, the sample size far exceeds the minimum number of responses/items required for quantitative studies.

According to the Portuguese Board of Nurses, there were 7383 nurses working in primary healthcare. Given that our sample consisted of 1059 nurses, we obtained a response rate of 14.3%.

### 2.3. Instrument

The Nurse Survey Instrument (Core Nurse Survey) of the RN4Cast Project (2018) was used for data collection, according to the structure defined by the international RN4Cast consortium, which was organized into four sections [8,27,30,31]:

- Section (A), which assesses the nursing practice environment using the Practice Environment Scale of Nursing Work Index (PES-NWI) [32] translated and validated by Amaral [33], nurses’ burnout using the Maslach Burnout Inventory translated and validated by Marques-Pinto [34], and other questions related to nurses’ work characteristics;
- Section (B), which assesses issues relating to the quality and safety of care;
- Section (C), which examines the organization of nurses’ work;
- Section (D), which characterizes the professionals socio-demographically.

The PES-NWI, with an alpha (α) of 0.89, consists of 31 items with answers on a 4-point Likert scale and has already been used in several studies [8,26,27,30,31,35]. The five dimensions of this scale are nursing participation in organization affairs, nursing foundations for quality of care, nurse manager ability, leadership, and support of nurses, staffing and resource adequacy, and collegial nurse–physician relations.

Burnout was assessed using the Maslach Burnout Inventory Human Services Survey [17], with an alpha of 0.87 and 22 items, which were also answered using a 7-point Likert scale that measures three components: emotional exhaustion, depersonalization, and lack of personal accomplishment [34].

The socio-demographic data considered were age, gender, academic qualifications, years in the profession, years in the current organization, years in the unit where they worked, primary healthcare as their only job, and satisfaction with the choice of nursing as a career.

### 2.4. Data Collection

The data were collected between November 2017 and May 2018 through the RN4Cast Portugal Project (2018), developed by the Portuguese Catholic University under the coordination of Prof Élvio Jesus. Nurses were asked to participate via a link to the online Nurse Survey Instrument, using Microsoft forms, which was widely publicized by the Portuguese Nurses Association. The questionnaire included an introductory note summarizing this study and its objectives, as well as ensuring the anonymity and confidentiality of responses and informed consent.

### 2.5. Data Analysis

Statistics were analyzed using IBM^®^-SPSS Statistics^®^ software version 28.0 and correlation methods. The normal distribution of the variables was analyzed using the skewness (Sk) and kurtosis (Ku) coefficients. An exploratory analysis of the variables was carried out, breaking down the measures of central tendency and dispersion. Categorical variables were described by absolute frequencies (n) and relative frequencies (%), while quantitative variables were described by the mean, standard deviation, minimum, and maximum. The statistical analysis was carried out using parametric tests, justified by the normality of the data distribution. Correlations between quantitative variables were analyzed using Pearson’s correlation test, and construct validity using Cronbach’s alpha coefficient. The significance level was 5%.

### 2.6. Ethical Issues

This study was conducted in accordance with the guidelines established by the Declaration of Helsinki [36]. The RN4Cast Portugal Project received a two favorable opinion from the Ethics Committee of the Porto Regional Centre of the Portuguese Catholic University: Ethics Clearance with approval date 3 July 2013 and a reconfirmation with Ethics Clearance number 03/2018 and date of approval 14 May 2018. It should be added that the nurses’ participation was voluntary and consensual and that this was stated and recorded by submitting the online form.

## 3. Results

### 3.1. Socio-Demographic and Professional Characteristics

The sample included 1059 primary healthcare nurses (Table 1) in Portugal, from fifty-five health center groups in mainland Portugal, fifteen health centers in the Autonomous Region of Madeira, and six health centers in the Autonomous Region of the Azores, representing around 14.3% of all nurses at this level of care in the Portuguese National Health Service.

The average age of the participants was 43.5 years (standard deviation (SD) = 7.9), with a predominance of females (85.8%). Most of the nurses (98.9%) had a bachelor’s degree and 54.7% were specialist nurses. The average length of professional practice was 20.5 years (SD = 7.8), with 14.5 years (SD = 10.0) in the current organization and 9.6 years (SD = 7.6) in the unit where they worked. For 83.2% of the nurses, primary healthcare was their only job, and 57.3% said they were satisfied or very satisfied with their choice of nursing as a career.

### 3.2. Job Satisfaction Among Nurses

The job satisfaction scale is a four-point Likert scale ranging from 1—very dissatisfied and 4—very satisfied. When asked about the “degree of satisfaction with their current role in the organization”, 49.5% of nurses reported being moderately satisfied, rising to 60.7% when including those who were very satisfied. The average recorded for this item was $\bar{x}$ = 2.6 (SD = 0.9), reflecting a moderate level of satisfaction. Regarding dissatisfaction, 39.3% of the nurses said they were dissatisfied with their current role in the organization.

Regarding the working environment, 79.1% of the participants rated it as reasonable to excellent, with an average of $\bar{x}$ = 2.2 (SD = 0.8), indicating a tendency to rate it as reasonable, and 20.9% considered their working environment in the organization to be poor. Regarding recommending their organization as a good place to work to other colleagues, 66.8% answered in the affirmative, with an average of $\bar{x}$ = 2.8 (SD = 0.8). In addition, 83.9% would recommend their organization to friends and family in the event of needing care, with an average of $\bar{x}$ = 3.1 (SD = 0.7). When asked about their satisfaction with various aspects, 75.0% of nurses said they were satisfied with flexible working hours, 82.2% with opportunities for advancement, 18.5% with job autonomy, 49.4% with professional status, 89.0% with salary, 54.4% with training opportunities, 40.4% with annual leave, 35.1% with sick leave, and 49.8% with study/training leave.

### 3.3. Nurses’ Intention to Leave

Regarding intention to leave, 59.7% of nurses said they had no intention of leaving the organization next year due to job dissatisfaction. Of the 427 who indicated the possibility of leaving, 45.4% would consider moving outside of the profession. The mean for this variable was $\bar{x}$ = 2.1 (SD = 0.9), suggesting a possible intention to leave for another workplace, but within nursing.

Regarding the ease of finding another acceptable job in the area, only 22.9% of nurses thought it would be very easy or relatively easy, while 77.1% believed it would be difficult or very difficult. The average for this variable was $\bar{x}$ = 1.9 (SD = 0.8), showing a generalized perception of difficulty in finding another job in the same area.

### 3.4. Burnout Among Nurses

With regard to burnout, the internal consistency of the dimensions of emotional exhaustion (symptoms of depletion of emotional resources in relation to work), depersonalization (adoption of a defensive, distanced, and cold attitude towards the client), and lack of personal accomplishment (development of feelings of ineffectiveness and lack of gratification in relation to work) were assessed, obtaining Cronbach’s alphas of 0.90, 0.73, and 0.78, respectively, showing very good and reasonable internal consistency (Table 2). Regarding burnout, 36.8% of nurses said that “I feel emotionally drained by my work”. The average value for this item was closer to “A few times a month” ($\bar{x}$ = 3.4, SD = 1.7).

The emotional exhaustion dimension had an average score of $\bar{x}$ = 2.8 (SD = 1.4) and was classified as moderate according to Marôco et al. [37], who, in turn, follow the recommendation of Maslach and Jackson [38]. We found four items with scores above 3 and five items with scores below 3. The highest average score in this dimension was obtained in item 2, “I feel worn out at the end of the working day”, while the lowest was obtained in item 6, “Working with people all day is really a strain on me”. This dimension proved to have very good internal consistency for this sample, with a Cronbach’s alpha of 0.90.

According to Vala et al. [39], the meanings of the items in the lack of personal accomplishment dimension should be inverted, so that higher values represent a decrease in professional achievement. Thus, as we obtained a low value, it means that the nurses who took part in this study had a lower lack of personal accomplishment.

### 3.5. Relationship Between the Nursing Practice Environment and Job Satisfaction, Intention to Leave, and Burnout

By analyzing the association between the variables in this study (Table 3), we can see that the higher the nursing practice environment value in primary healthcare, the higher the rating of job satisfaction, which has a moderate association (r = 0.60), and the lower the level of burnout, with a weak and negative association (r = −0.28), especially in the emotional exhaustion dimension, where there was a weak and negative association (r = −0.35).

On the other hand, when analyzing the association between the variables job satisfaction and burnout, the higher the value of job satisfaction in primary healthcare, the lower the levels of burnout, with a weak and negative association between them (r = −0.23). This was further corroborated in the burnout dimensions, where there were negative, low, and very low correlations (emotional exhaustion r = −0.31; depersonalization r = −0.11; lack of personal accomplishment r = −0.22).

Regarding intention to leave, there was no statistically significant correlation.

When we analyzed the association of the nursing practice environment dimensions (nursing participation in organization affairs, nursing foundations for quality of care, nurse manager ability, leadership, and support of nurses, staffing and resource adequacy, and collegial nurse–physician relations with job satisfaction), we found weak and low correlations. More specifically, there were weak associations between job satisfaction and nursing foundations for quality of care, nurse manager ability, leadership, and support of nurses, and collegial nurse–physician relations, with *r* values of 0.30, 0.38, and 0.39, respectively.

On the subject of the nursing practice environment, the weakest correlation between its dimensions (Table 4) was between collegial nurse–physician relations and nurse manager ability, leadership, and support of nurses, with a value of r = 0.27, while the strongest correlation, which was moderate, was between nursing participation in organization affairs and nurse manager ability, leadership, and support of nurses, with a value of r = 0.63. The nursing practice environment dimensions showed positive correlations with each other, with the highest correlation being between nurse manager ability, leadership, and support of nurses and nursing participation in organization affairs (r = 0.63), followed by nursing foundations for quality of care (r = 0.46).

The lowest correlation was recorded between nurse manager ability, leadership, and support of nurses and collegial nurse–physician relations (r = 0.27), followed by staffing and resource adequacy (r = 0.30).

The observed *p*-values of less than 0.01 and 0.05 in Table 3 and Table 4 indicate statistical significance in the correlation indices.

Table 5 shows that nurses perceive the nursing practice environment in primary healthcare in Portugal as unfavorable and mixed, associated with low levels of job satisfaction, moderate intention to leave, and low levels of burnout.

## 4. Discussion

The results obtained in this study indicate that the participating nurses perceive an unfavorable and mixed nursing practice environment in primary healthcare in Portugal [1], with nurse participation in organization affairs averaging 2.2, nurse manager ability, leadership, and support of nurses averaging 2.4, staffing and resource adequacy averaging 2.4, and the total of the nursing practice environment averaging 2.5. These results are associated with moderate job satisfaction, moderate intentions to leave, and a low level of burnout. An analysis of the burnout dimensions revealed moderate emotional exhaustion, low depersonalization, and low lack of personal accomplishment.

The results of this study suggest that a positive nursing practice environment is strongly associated with higher levels of job satisfaction and lower burnout, corroborating the conclusions of Nantsupawat et al. [19].

The influence of the nursing practice environment on job satisfaction in this study was relevant, as demonstrated in the studies by Ayamolowo et al. [40] and Klopper et al. [8]. Abraham et al. [41] identified that a favorable nursing practice environment correlates with high levels of job satisfaction, while Yasin et al. [42] reported an association between management support and job satisfaction.

This study found a negative correlation between the nursing practice environment and the dimensions of burnout—emotional exhaustion, depersonalization, and lack of personal accomplishment. In other words, the higher the nursing practice environment value in primary healthcare, the lower the burnout, with a weak and negative association, especially in the emotional exhaustion dimension. These findings are consistent with those of Wang et al. [20] and Coetzee et al. [30], who reported a negative relationship between the dimensions of the nursing practice environment and those of burnout, with the latter being stronger with emotional exhaustion, as was the case in this study. This fact is also reaffirmed and reinforced in the study by Abraham et al. [41], who concluded that a favorable nursing practice environment has the potential to reduce burnout among primary healthcare nurses.

Considering that the nursing practice environment is one of the variables that influence nurses’ job satisfaction, the unfavorable and mixed rating of primary healthcare in Portugal is a worrying issue that requires attention and needs to be addressed and worked on to promote the development of continuous improvement measures in this care context [1]. It is therefore considered to be vital information for the nurse managers, administrators, and nursing directors of health institutions and for policymakers, since the promotion and creation of measures to favor favorable primary healthcare is vital to guarantee the satisfaction of both nursing professionals and clients, resulting in an overall improvement in the quality of nursing care [1]. Within the framework of health policies, it is important to promote measures that value human resources, such as creating incentives to retain experienced professionals who are committed to the organization, creating management and leadership training programs for nurse managers, assessing leadership skills with regular feedback, and redesigning workflows to reduce overload and the duplication of tasks.

According to Lucas et al. [1], nurses perceive that they are satisfied with the provision of care, with a focus on quality and multidisciplinary relationships. However, they consider the dimensions related to management support and performance, the exercise of leadership, and participation in the organization’s governance to be unfavorable [1]. The adequacy of resources was also considered unfavorable, which conveys a worrying reality about allocations that significantly impact outcomes for clients and nurses [1].

Regarding job satisfaction, 39.3% of the nurses said they were dissatisfied with their current role in the organization and 20.9% considered their working environment in the organization to be poor. We found a higher figure than in the study by Coetzee et al. [30], who obtained 32.2% in a hospital context regarding dissatisfaction with their current role in the organization and the same figure for the classification of a poor working environment. The reasons most cited as unsatisfactory by nurses were a lack of opportunities for progression, with an average of 1.7, salary issues, with an average of 1.4, training opportunities, with 54.4 percent, and study/training leave, with an average of 2.0. In the study by Aiken et al. [43], the proportion of nurses dissatisfied with their current role in the organization was between 38.3% and 48.1% in Scotland, England, Columbia, and Pennsylvania.

On the question of whether the primary healthcare nurses who took part in this study would recommend their organization to another nurse colleague as a good place to work, 33.3% answered no, and 16.0% of nurses would not recommend their organization to their friends and family if they needed care, results that are identical to those of the study by Coetzee et al. [30].

As far as dissatisfaction with salary is concerned, this was very significant and higher than the 75.6% of nurses in Shanghai hospitals reported in the study by You et al. [35].

The variable of job satisfaction showed a positive association with the nursing practice environment and a negative association with burnout and its dimensions: emotional exhaustion, depersonalization, and lack of personal accomplishment. These data are identical to the results of Coetzee et al. [30], where the correlation between job satisfaction and the emotional exhaustion and depersonalization dimensions was negative, being stronger with emotional exhaustion than depersonalization, which is what happened in this study.

The nurses who took part in this study showed low job satisfaction, with a mean of $\bar{x}$ = 2.0 (SD = 0.4), which is in line with the results of the studies by Ayamolowo et al. [40] on primary healthcare in Nigeria, Khamisa et al. (2015) [18] on hospitals in South Africa, Nantsupawat et al. [44] on hospitals in Thailand, and You et al. [35] on hospitals in Shanghai. In contrast, nurses working in primary healthcare in Massachusetts showed high job satisfaction, as described by Poghosyan et al. [45].

We found that nurses reported moderate job satisfaction within primary healthcare in Portugal, which, being a relevant management indicator, requires great attention from the nurse managers, administrators, and nursing boards of health institutions. This study showed that the participation of nurses in the governance of the organization and the adequacy of human and material resources were the dimensions that most positively influenced job satisfaction. Issues related to salary and opportunities for progression were identified as the areas of greatest dissatisfaction. In our sample, 54.7% of whom were specialist nurses, these issues of salary and opportunities for progression were particularly relevant, given that from 2009 to the date of data collection, there had been no open competition for nurse managers. This situation can be perceived by the nurses who took part in this study as a great professional injustice, especially considering the many nurse managers who have left organizations to retire and the use that has been made, at little cost, of the work of nurses and nurse specialists to perform the duties of nurse managers. Although this practice can lead to controlled costs in the short term with nursing professionals, it does not contribute to the creation of long-term value for organizations. As Carvalho et al. [46] point out in their study, the main responsibility of nurse managers is to ensure the quality of nursing care in the teams and health services that they manage. It is therefore essential to have managers who are committed to and motivated by their work. As Porter [47] points out, value in healthcare is measured by the results achieved and not by the volume of services provided, so shifting the focus from volume to value is a central challenge for improving the quality and efficiency of the care provided. As value is defined as cost-related results, it encompasses efficiency [47]. Reducing costs without considering the results achieved is dangerous and self-defeating, leading to false “savings” that can potentially limit the effective delivery of healthcare [47]. The “false economies” of not replacing nurse managers and specialists can compromise “value creation” in National Health Service organizations. Another related issue is the fact that there are nurses with specialized clinical training, many of whom have been given the title of specialist nurse by the Order of Nurses, and there is an urgent need to open competition for new specialist nurses and nurse managers.

Studies such as those by Cagan and Gunay [48], Khamisa et al. [18], and Nantsupawat et al. [19] describe the importance that personal satisfaction has on job satisfaction. It seems to us that the high percentage of dissatisfaction with the choice of nursing as a career in this study is not unrelated at 42.7%, which is higher than the previous RN4Cast study in a hospital setting, which had a dissatisfaction rate of 40.3% [49]. It should be stressed that specialist nurses are, overall, a sub-group of professionals who are committed to professional development and who should be involved in any intention to organize and develop services and functional units in the ACES. They are an important resource that nurse managers can and should turn to. Scientific evidence clearly demonstrates the importance of investing in training, highlighting the positive relationship between increased training and an improved nursing practice environment, quality, and safety of care, as well as the empowerment of human resources [1,26,35,50,51,52,53].

In the intention to leave variable, 40.3% of nurses would leave their current organization in the next year due to job dissatisfaction, contrary to the results of the study by Callado et al. [54], which was also conducted with primary healthcare nurses in Portugal, where only 13.2% of nurses intended to leave. In this situation, what may explain this difference in the nurses’ intention to leave is related to the period of data collection when a new nursing career was already implemented, which emphasizes advanced nursing and nursing management. The results of the present study are also corroborated by those of the larger study by Aiken et al. (2012) of nurses in Belgium, Germany, Sweden, and Switzerland, which are lower than the results in Finland, Greece, Ireland, and Poland and higher than those in the Netherlands, Norway, Spain, and the USA [26]. Of these, 45.4% would leave nursing. Although this figure is lower than the 54.4% reported in the study by Coetzee et al. [30], we believe that the departure of nurses could compromise the quality of care through human resource allocations, with a negative impact on organizations [55].

On the other hand, the study by Yasin et al. [42] in Canadian hospitals showed a negative association between intention to leave and job satisfaction. Similarly, the study by Poghosyan et al. [55] in primary healthcare in the state of Massachusetts showed that a favorable nursing practice environment promotes job satisfaction and reduces nurses’ intention to leave. The study by Marques-Pinto et al. [56] also agrees with this relationship, indicating that the greater the degree of participation nurses have in the organization’s responsibilities, the greater their intention to stay. The possibility of making decisions that impact their professional practice and environment is an important motivational factor for nurses [56].

In the present study, nurses showed a moderate intention to leave (40.3%), and 77.1% considered it difficult to find an acceptable job in nursing if they were looking for another job. The greater the degree to which nurses are involved in the organization’s affairs, the lower their exhaustion and the lower their intention to leave the organization. This is because the possibility of making decisions that impact their professional practice and environment is an important motivating factor for nurses [56].

The labor context in the country at the time of this study’s data collection, regarding the non-recognition of specialized nursing intervention as a career coupled with the fact that our sample was made up mostly of specialist nurses with an average career of 20.5 years, may be motivating factors for the intention to leave variable.

In the study by Callado et al. [54], which was carried out after changes to legislation and nursing as a career, nurses reported decreased intentions to leave primary healthcare, with 78.0% being very satisfied with having chosen to work in primary healthcare. The changes to nursing as a career included the category of specialist nurse, allowing many nurses to move to this new category and obtain a salary increase, which may justify a greater intention to remain in the organization [54,57].

Regarding burnout, there was a greater association in the opposite direction with staffing and resource adequacy, the nursing practice environment, and job satisfaction. The burnout variable was only positively associated with its emotional exhaustion dimension, followed by depersonalization and lack of personal accomplishment. When we analyzed the emotional exhaustion dimension, we found a greater association, in the opposite direction, with staffing and resource adequacy, similar to Bruyneel et al. [51]. These results are in line with the theory of Demerouti and Bakker [58] and Schaufeli and Bakker [59] on the negative relationship between work resources and burnout. Marques-Pinto et al. [56] also expand on these results, as they tested the mediating role of burnout in the relationship between work resources, nurses’ participation in the organization’s affairs, and intention to leave.

On the other hand, a study on primary healthcare nurses in Pennsylvania found a relationship between burnout and reduced quality of care [51]. Khamisa et al. [18] concluded that burnout has a clear impact on the mental health and well-being of nurses, which is likely to jeopardize productivity, performance, and the quality of client care.

## 5. Limitations and Recommendations

This study has limitations similar to other investigations carried out in health services using cross-sectional data. The association identified in the sample may not be representative of the general population, which restricts the ability to infer causality. In addition, collecting data by self-report may introduce bias into the responses.

Although a nationally representative sample was obtained, covering a significant variety of territories within primary healthcare, including the Autonomous Regions of Madeira and the Azores, the response rate was only 14.3%, which is a limitation of this study and may be associated with the method of data collection, which was carried out online. This low response rate could probably impact the representativeness of this study’s results, although without detracting from its importance. The COVID-19 pandemic has accelerated the use of digital questionnaires, which may have led to a lack of interest in answering on the part of nurses.

Future studies could adopt other methodological approaches, such as mixed-methods studies that integrate quantitative and qualitative analyses, which could deepen our understanding of the factors that influence nurses’ job satisfaction. Studies using qualitative data, through interviews or focus groups, could reveal subjective perceptions and institutional dynamics that have not yet been explored.

Furthermore, the interrelationships observed between the variables in this study suggest that they are influenced by factors that go beyond recent or temporary trends in healthcare. If this were not the case, the problem analyzed would already have been resolved, which unfortunately is not the case. We also identified a lack of relevant research exploring the nursing practice environment in primary healthcare.

We also propose the development of intervention studies with a quasi-experimental or experimental design that assess the impact of measures such as improving nursing practice environments, recognition programs, and salary reviews on job satisfaction and retention. Longitudinal studies can contribute to understanding the evolution of these indicators over time. Finally, we would emphasize the importance of integrating the views of managers and users, as well as exploring the potential of digital technologies and big data for continuous monitoring of satisfaction and quality in nursing practice environments.

## 6. Conclusions

Optimizing the nursing practice environment in healthcare institutions is a cost-effective organizational approach that aims to achieve improved outcomes for both clients and nurses. This includes promoting high professional performance and improving the quality of nursing care.

We believe that this study is relevant because it will enable nurses, managers, and political decision-makers responsible for health policies to reorient their practices so that they can base their decisions on scientific evidence, especially with regard to creating conditions conducive to a favorable nursing practice environment.

There was a higher correlation between the nursing practice environment and job satisfaction and a negative correlation with burnout and its dimensions: emotional exhaustion, depersonalization, and lack of personal accomplishment.

Regarding intention to leave, 40.3% of primary healthcare nurses would leave their current organization due to professional dissatisfaction. The departure of nurses can compromise the quality of care through human resource allocations, with a negative impact on organizations.

Burnout was more strongly associated, in the opposite direction, with staffing and resource adequacy, the nursing practice environment, and job satisfaction.

The primary healthcare nurses in Portugal who took part in this study had a low level of burnout, with moderate emotional exhaustion, low depersonalization, and low lack of personal accomplishment. The primary healthcare setting is more favorable than the hospital setting in terms of this variable. Regarding strategies to promote job satisfaction and burnout among nurses, they can be organized into organizational, individual, professional development, and health policy strategies. Organizational strategies can include adjusting the nurse/patient ratio, ensuring balanced working hours and adequate breaks, promoting authentic and empathetic leadership with strong support from managers, and creating favorable working environments. Individual strategies can include psychological support, promoting relaxation techniques (mindfulness, breathing, etc.), and encouraging work–life balance. Professional development strategies could include investing in continuous training and clinical and advanced specializations, creating career plans and progression opportunities that are more appropriate for a highly demanding and fast-paced profession, and involving nurses in decision-making. As part of health policies, it is essential to enhance nursing as a career (roles, salaries, and contracts), increase public investment in human resources for health, and implement policies to retain and value professionals.

Thus, improving the nursing practice environment in healthcare organizations is a low-cost organizational strategy that can generate favorable results for clients and promote the retention of qualified nurses, thus contributing to raising the quality of nursing care and the efficiency of the healthcare system.

## Figures and Tables

**Table 1:** Descriptive analysis of socio-demographic data.

	N	%	$\bar{x}$	SD
Gender				
Female	909	85.8		
Male	150	14.2		
Academic Degree				
Bachelor’s	1047	98.9		
Specialized				
Specialist nurses	579	54.7		
Do you work in other places?				
Yes	178	16.8		
No	881	83.2		
Satisfaction with choosing nursing as a career				
Very dissatisfied	180	17.0		
Not satisfied	272	25.7		
Satisfied	364	34.4		
Very Satisfied	243	22.9		
Age			43.5	7.9
Years in the profession			20.5	7.8
Years in current organization			14.5	10.0
Years in current Unit/Service			9.6	7.6

$\bar{x}$—Average. SD—Standard Deviation.

**Table 2:** Descriptive results of the burnout dimensions (n = 1059).

Dimensions	$\bar{x}$	SD	α
Emotional exhaustion	2.8	1.4	0.90
Depersonalization	0.9	1.0	0.73
Lack of Personal Accomplishment	1.2	0.8	0.78

$\bar{x}$—Average. SD—Standard Deviation. α—Cronbach’s alpha.

**Table 3:** Correlations of variables.

	Professional Satisfaction	Burnout—Total	Burnout—Emotional Exhaustion	Burnout—Depersonalization	Burnout—Lack of Personal Accomplishment
Nursing Participation in Organization Affairs	0.55 **	−0.24 **	−0.30 **	−0.13 **	−0.11 **
Nurse Manager Ability, Leadership, and Support of Nurses	0.38 **	−0.21 **	−0.26 **	−0.11 **	−0.09 **
Nursing Foundations for Quality of Care	0.39 **	−0.12 **	−0.17 **	−0.17 **	−0.14 **
Staffing and Resource Adequacy	0.40 **	−0.29 **	−0.36 **	−0.11 **	−0.10 **
Collegiate Nurse–Physician Relations	0.30 **	−0.13 **	−0.18 **	−0.11 **	−0.12 **
Nursing Practice Environment	0.60 **	−0.28 **	−0.35 **	−0.18 **	−0.15 **
Job Satisfaction	1	−0.23 **	−0.31 **	−0.11 *	−0.22 *
*Total Burnout*		1	0.88 **	0.55 **	0.13 **
*Burnout*—Emotional Exhaustion			1	0.40 **	0.22 **
*Burnout*—Depersonalization				1	0.31 **

* *p* < 0.05; ** *p* < 0.01.

**Table 4:** Correlation between nursing practice environment variables.

	NMALSN	NFQC	SRA	CNPR	Nursing Practice Environment (NPE)
Nursing Participation in Organization Affairs (NPOA)	0.63 **	0.46 **	0.43 **	0.41 **	0.90 **
Nurse Manager Ability, Leadership, and Support of Nurses (NMALSN)	1	0.39 **	0.30 **	0.27 **	0.74 **
Nursing Foundations for Quality of Care (NFQC)		1	0.33 **	0.45 **	0.70 **
Staffing and Resource Adequacy (SRA)			1	0.32 **	0.62 **
Collegiate Nurse–Physician Relations (CNPR)				1	0.59 **

** *p* < 0.01.

**Table 5:** Descriptive data for the variables.

Variables	$\bar{x}$	SD
PES/NWI	2.5	0.4
Job Satisfaction	2.0	0.4
Burnout	1.6	0.8
	%	N
Intention to Leave	40.3	427

$\bar{x}$—Average. SD—Standard Deviation.

## Data Availability

Restrictions apply to the availability of these data. Data were obtained from a third party and are available with the permission of the third party.

## Public Involvement Statement

No public involvement in any aspect of this research.

## Guidelines and Standards Statement

This manuscript was drafted against the Strengthening the Reporting of Observational Studies in Epidemiology (STROBE) Statement: Guidelines for reporting observational studies [60].

## References

[1] Lucas P., Jesus É., Almeida S., Araújo B. (2023). Relationship of the nursing practice environment with the quality of care and patients’ safety in primary health care. BMC Nurs..

[2] Lake E. (2007). The nursing practice environment. Med. Care. Res. Rev..

[3] Gensimore M., Maduro R., Morgan M., McGee G., Zimbro K. (2020). The effect of nurse practice environment on retention and quality of care via burnout, work characteristics, and resilience. J. Nurs. Admin..

[4] Twigg D., McCullough K. (2014). Nurse retention: A review of strategies to create and enhance positive practice environments in clinical settings. Int. J. Nurs. Stud..

[5] Lambrou P., Merkouris A., Middleton N., Papastavrou E. (2014). Nurses’ perceptions of their professional practice environment in relation to job satisfaction: A review of quantitative studies. Health Sci. J..

[6] International Council of Nurses (2014). Nuses: A Force for Change-A Vital Resource for Health: International Nurses Day 2014.

[7] Aiken L., Cimiotti J., Sloane D., Smith H., Flynn L., Neff D. (2011). Effects of nurse staffing and nurse education on patient deaths in hospitals with different nurse work environments. Med. Care..

[8] Klopper H., Coetzee S., Pretorius R., Bester P. (2012). Practice environment, job satisfaction and burnout of critical care nurses in South Africa. J. Nurs. Manag..

[9] Lucas P., Jesus E., Almeida S., Araújo B. (2021). Validation of the psychometric properties of the practice environment scale of nursing work index in primary health care in Portugal. Int. J. Environ. Res. Public. Health..

[10] Falguera C.C., De los Santos J.A.A., Galabay J.R., Firmo C.N., Tsaras K., Rosales R.A., Mirafuentes E.C., Labrague L.J. (2021). Relationship between nurse practice environment and work outcomes: A survey study in the Philippines. Int. J. Nurs. Pract..

[11] Boudreau C., Rhéaume A. (2024). Impact of the work environment on nurse outcomes: A mediation analysis. West. J. Nurs. Res..

[12] Labrague L.J., Al Sabei S., AbuAlRub R., Burney I., Al Rawajfah O. (2025). The role of nurses’ adherence to clinical safety guidelines in linking nurse practice environment to missed nursing care. J. Nurs. Scholarsh..

[13] Bautista J.R., Lauria P.A.S., Contreras M.C.S., Maranion M.M.G., Villanueva H.H., Sumaguingsing R.C., Abeleda R.D. (2020). Specific stressors relate to nurses’ job satisfaction, perceived quality of care, and turnover intention. Int. J. Nurs. Pract..

[14] Kowalski M., Basile C., Bersick E., Cole D., McClure D., Weaver S. (2020). What do nurses need to practice effectively in the hospital environment? An integrative review with implications for nurse leaders. Worldviews. Evid. Based. Nurs..

[15] Salahat M.F., Al-Hamdan Z.M. (2022). Quality of nursing work life, job satisfaction, and intent to leave among Jordanian nurses: A descriptive study. Heliyon.

[16] Wong C., Laschinger H. (2015). The influence of frontline manager job strain on burnout, commitment and turnover intention: A cross-sectional study. Int. J. Nurs. Stud..

[17] Maslach C., Leiter M. (2016). Understanding the burnout experience: Recent research and its implications for psychiatry. World Psychiatry.

[18] Khamisa N., Oldenburg B., Peltzer K., Ilic D. (2015). Work related stress, burnout, job satisfaction and general health of nurses. Int. J. Environ. Res. Public. Health..

[19] Nantsupawat A., Kunaviktikul W., Nantsupawat R., Wichaikhum A., Thienthong H., Poghosyan L. (2017). Effects of nurses work environment on job dissatisfaction, burnout, intention to leave. Int. Nurs. Rev..

[20] Wang S., Liu Y., Wang L. (2015). Nurse burnout: Personal and environmental factors as predictors. Int. J. Nurs. Pract..

[21] Laschinger H., Leiter M. (2006). The impact of nursing work environments on patient safety outcomes. J. Nurs. Admin..

[22] Costa P., Sousa J.P., Nascimento T., Cruchinho P., Nunes E., Gaspar F., Lucas P. (2025). Leadership Development in Undergraduate Nursing Students: A Scoping Review. Nurs. Rep..

[23] Martínez-Riera J.R., Juárez-Vela R., Díaz-Herrera M.Á., Montejano-Lozoya R., Doménech-Briz V., Benavent-Cervera J.V., Cabellos-García A.C., Melo P., Nguyen T.H., Gea-Caballero V. (2020). Qualitative Analysis by Experts of the Essential Elements of the Nursing Practice Environments Proposed by the TOP10 Questionnaire of Assessment of Environments in Primary Health Care. Int. J. Environ. Res. Public Health.

[24] Poghosyan L., Shang J., Liu J., Poghosyan H., Liu N., Berkowitz B. (2015). Nurse practitioners as primary care providers. Health. Care. Manage Rev..

[25] Rabie T., Klopper H., Coetzee S. (2017). Creating positive practice environments in a primary health care setting. Int. J. Nurs. Pract..

[26] Aiken L., Sermeus W., Van den Heede K., Sloane D., Busse R., McKee M., Bruyneel L., Rafferty A.M., Griffiths P., Tishelman C. (2012). Patient safety, satisfaction, and quality of hospital care: Cross sectional surveys of nurses and patients in 12 countries in Europe and the United States. BMJ.

[27] Sermeus W., Aiken L., Van den Heede K., Rafferty A., Griffiths P., Moreno-Casbas M., Busse R., Londqvist R., Scott A.P., Bruyneel l. (2011). Nurse forecasting in Europe (RN4CAST): Rationale, design and methodology. BMC Nurs..

[28] von Elm E., Altman D.G., Egger M., Pocock S.J., Gøtzsche P.C., Vandenbroucke J.P. (2007). The strengthening the reporting of observational studies in epidemiology (STROBE) statement: Guidelines for reporting observational studies. Lancet.

[29] Marôco J. (2021). Análise Estatística com o SPSS Statistics.

[30] Coetzee S., Klopper H., Ellis S., Aiken L. (2013). A tale of two systems-Nurses practice environment, well being, perceived quality of care and patient safety in private and public hospitals in South Africa: A questionnaire survey. Int. J. Nurs. Stud..

[31] Squires A., Aiken L., van den Heede K., Sermeus W., Bruyneel L., Lindqvist R., Schoonhoven L., Stromseng I., Busse R., Brzostek T. (2013). A systematic survey instrument translation process for multi-country, comparative health workforce studies. Int. J. Nurs. Stud..

[32] Lake E. (2002). Development of the practice environment scale of the nursing work index. Res. Nurs. Health..

[33] Amaral A., Ferreira P., Lake E. (2012). Validation of the practice environment scale of the nursing work index (pes-nwi) for the portuguese nurse population. Int. J. Caring. Sci..

[34] Jesus É., Pinto A., Fronteira I., Mendes A. (2014). Estudo RNACAST em Portugal: Percepção dos enfermeiros sobre burnout. Rev. Invest Enf..

[35] You L., Aiken L., Sloane D., Liu K., He G., Hu Y., Jiang X., Li X., Liu H., Shang S. (2013). Hospital nursing, care quality, and patient satisfaction: Cross-sectional surveys of nurses and patients in hospitals in China and Europe. Int. J. Nurs. Stud..

[36] World Medical Association (2013). Worldmedical association declaration of Helsinki ethical principles for medical research involving human subjects. Clin. Rev. Educ..

[37] Marôco J., Marôco A., Leite E., Bastos C., Vazão M., Campos J. (2016). Burnout in portuguese healthcare professionals: An analysis at the national level. Acta. Med. Port.

[38] Maslach C., Jackson S. (1985). The role of sex and family variables in burnout. Sex Roles.

[39] Vala J., Pinto A., Moreira S., Lopes R., Januário P. (2017). Burnout na Classe Médica em Portugal: Perspetivas Psicológicas e Psicossociológicas.

[40] Ayamolowo S., Irinoye O., Oladoyin M. (2013). Job satisfaction and work environment of primary health care nurses in Ekiti State, Nigeria: An exploratory study. Int. J. Caring. Sci..

[41] Abraham C., Zheng K., Norful A., Ghaffari A., Liu J., Poghosyan L. (2021). Primary care nurse practitioner burnout and perceptions of quality of care. Nurs. Forum.

[42] Yasin Y., Kerr M., Wong C., Bélanger C. (2020). Factors affecting job satisfaction among acute care nurses working in rural and urban settings. J. Adv. Nurs..

[43] Aiken L., Clarke S., Sloane D. (2002). Hospital staffing, organization, and quality of care: Cross-national findings. Int. J. Qual. Health Care.

[44] Nantsupawat A., Srisuphan W., Kunaviktikul W., Wichaikhum O., Aungsuroch Y., Aiken L.H. (2011). Impact of nurse work environment and staffing on hospital nurse and quality of care in Thailand. J. Nurs. Scholarsh..

[45] Poghosyan L., Liu J., Shang J., D’Aunno T. (2016). Practice environments and job satisfaction and turnover intentions of nurse practitioners. Health. Care. Manage Rev..

[46] Carvalho M., Gaspar F., Potra T., Lucas P. (2022). Translation, Adaptation, and Validation of the Self-Efficacy Scale for Clinical Nurse Leaders for the Portuguese Culture. Int. J. Environ. Res. Public. Health..

[47] Porter M. (2010). What is value in health care?. N. Engl. J. Med..

[48] Cagan O., Gunay O. (2015). The job satisfaction and burnout levels of primary care health workers in the province of Malatya in Turkey. Pak. J. Med. Sci..

[49] Roque S. (2016). Impacto do Ambiente de Prática de Enfermagem na Qualidade e Segurança dos Cuidados.

[50] Aiken L., Clarke S., Sloane D., Lake E., Cheney T. (2008). Effects of hospital care environment on patient mortality and nurse outcomes. J. Nurs. Adm..

[51] Bruyneel L., Heede K., Diya L., Aiken L., Sermeus W. (2009). Predictive validity of the international hospital outcomes study questionnaire: An RN4CAST pilot study. J. Nurs. Scholarsh..

[52] Kirwan M., Matthews A., Scott P. (2013). The impact of the work environment of nurses on patient safety outcomes: A multi-level modelling approach. Int. J. Nurs. Stud..

[53] Titlestad I., Haugstvedt A., Igland J., Graue M. (2018). Patient safety culture in nursing homes: A cross-sectional study among nurses and nursing aides caring for residents with diabetes. BMC Nurs..

[54] Callado A., Teixeira G., Lucas P. (2023). Turnover intention and organizational commitment of primary healthcare nurses. Healthcare..

[55] Poghosyan L., Norful A., Martsolf G. (2017). Primary Care Nurse Practitioner Practice Characteristics. J. Ambul. Care. Manag..

[56] Marques-Pinto A., Jesus É., Mendes A., Fronteira I., Roberto M. (2018). Nurses’ intention to leave the organization: A mediation study of professional burnout and engagement. Span. J. Psychol..

[57] Santos L., Soares T. (2024). Impacto da remuneração dos enfermeiros na qualidade dos cuidados de saúde. Rev. Portug. Gestão. Saúde.

[58] Demerouti E., Bakker A. (2011). The job demands–resources model: Challenges for future research. SA J. Indust. Psychol..

[59] Schaufeli W., Bakker A. (2004). Job demands, job resources, and their relationship with burnout and engagement: A multi-sample study. J. Organ. Behav..

[60] von Elm E., Altman D.G., Egger M., Pocock S.J., Gøtzsche P.C., Vandenbroucke J.P., STROBE Initiative (2008). Strengthening the Reporting of Observational Studies in Epidemiology (STROBE) statement: Guidelines for reporting observational studies. J. Clin. Epidemiol..

